# Efficient Assembly and Secretion of Recombinant Subviral Particles of the Four Dengue Serotypes Using Native prM and E Proteins

**DOI:** 10.1371/journal.pone.0008325

**Published:** 2009-12-15

**Authors:** Pei-Gang Wang, Mateusz Kudelko, Joanne Lo, Lewis Yu Lam Siu, Kevin Tsz Hin Kwok, Martin Sachse, John M. Nicholls, Roberto Bruzzone, Ralf M. Altmeyer, Béatrice Nal

**Affiliations:** 1 Hong Kong University-Pasteur Research Centre, The University of Hong Kong, Hong Kong, China; 2 Department of Pathology, The University of Hong Kong, Hong Kong, China; 3 Department of Anatomy, The University of Hong Kong, Hong Kong, China; 4 Plate-Forme de Microscopie Ultrastructurale, Institut Pasteur, Paris, France; The University of Chicago, United States of America

## Abstract

**Background:**

Flavivirus infected cells produce infectious virions and subviral particles, both of which are formed by the assembly of prM and E envelope proteins and are believed to undergo the same maturation process. Dengue recombinant subviral particles have been produced in cell cultures with either modified or chimeric proteins but not using the native forms of prM and E.

**Methodology/Principal Findings:**

We have used a codon optimization strategy to obtain an efficient expression of native viral proteins and production of recombinant subviral particles (RSPs) for all four dengue virus (DV) serotypes. A stable HeLa cell line expressing DV1 prME was established (HeLa-prME) and RSPs were analyzed by immunofluorescence and transmission electron microscopy. We found that E protein is mainly present in the endoplasmic reticulum (ER) where assembly of RSPs could be observed. Biochemical characterization of DV1 RSPs secretion revealed both prM protein cleavage and homodimerization of E proteins before their release into the supernatant, indicating that RSPs undergo a similar maturation process as dengue virus. Pulse chase experiment showed that 8 hours are required for the secretion of DV1 RSPs. We have used HeLa-prME to develop a semi-quantitative assay and screened a human siRNA library targeting genes involved in membrane trafficking. Knockdown of 23 genes resulted in a significant reduction in DV RSP secretion, whereas for 22 others we observed an increase of RSP levels in cell supernatant.

**Conclusions/Significance:**

Our data describe the efficient production of RSPs containing native prM and E envelope proteins for all dengue serotypes. Dengue RSPs and corresponding producing cell lines are safe and novel tools that can be used in the study of viral egress as well as in the development of vaccine and drugs against dengue virus.

## Introduction

Dengue is one of the most important vector-borne viral diseases in humans. However, the interaction between dengue virus (DV) and host cells is only partly understood. Therefore, there is an urgent need to develop new tools to gain insight into the viral journey through host cells.

As a member of the *Flavivirus* genus in the *Flaviridae* family, DV is a small, positive strand RNA enveloped virus. There are four serotypes of dengue virus (DV1-4). Their genome encodes a polyprotein precursor of at least seven non-structural proteins and three structural proteins which are the capsid protein (C), the membrane protein (M) and the envelope glycoprotein (E) [1]. The polyprotein is processed co- and post-translationally by cellular signalase in the lumen of the rough endoplasmic reticulum (ER) and by a viral protease in the cytosol [1], [2], [3]. The nascent C protein contains a C-terminal hydrophobic domain that acts as a signal sequence for translocation of the immature form of M, the prM, into the lumen of the rough ER. Two adjacent prM C-terminal transmembrane domains are responsible for prM membrane anchoring and E translocation into the ER [2]. prM and E associate into heterodimers at ER membranes [4], [5] where they assemble with the viral RNA/C complex to form progeny virions [1]. During the egress of virions through the secretory pathway, prM protein is cleaved by the trans-Golgi resident furin protease to form the M envelope protein and the soluble pr segment, which is released into the extracellular medium upon particle secretion [6]. prM cleavage marks maturation of flavivirus virions [7], [8]. Cleavage of prM is intimately correlated to change of conformation of envelope protein complexes. Although it was thought that prM cleavage is a prerequisite for E dimerization, recent studies show that change of conformation most probably occurs at low pH in the TGN and allows cleavage of prM by furin [6], [9], [10].

prM and E proteins from flaviviruses, such as yellow fever virus [11], Japanese encephalitis virus (JEV) [12], [13], West Nile virus (WNV) [14] and tick-bone encephalitis virus (TBEV) [15], [16], are able to assemble into subviral particles in the absence of any other viral component. Subviral particles and infectious virions are co-produced in infected cells, assemble in an immature form, and subsequently undergo the same maturation process and display similar fusion activity as infectious viruses [17], [18]. Therefore, subviral particles could be a precious tool for research on cell biology of DVs. Although there have been attempts by several groups to obtain DV RSPs, either their production was inefficient or the sequence of DVs structural proteins had to be substantially modified [19] before they could efficiently generate RSPs. For example, the furin cleavage site on prM had to be mutated to establish the DV2 RSPs producing CHO cell line because it was found that the DV2 RSPs cause cell-cell fusion [20], [21]. Others replaced a portion of the carboxy-terminal region of DV1 and DV2 E genes with the corresponding sequence of JEV to observe significant RSP secretion [22], [23]. In either case, these modifications may interfere with the interactions with cytosolic proteins and, possibly, with the maturation and folding of the structural proteins.

Here we describe the efficient production of native RSPs of the four DV serotypes by using a codon optimization strategy. Codons of the DV prME gene were replaced with those preferentially used in mammalian cells. Optimization was first applied to the DV1 prME gene. We found that this experimental approach could efficiently increase the intracellular expression level of the E glycoprotein in human cells and therefore enhance the production of DV1 RSPs. We have established a DV1 RSPs producing stable cell line (HeLa-prME). Our data by immunofluorescence and electron microscopy show that most of E protein co-localizes with markers of the ER, where RSPs accumulate. Biochemical analysis of secreted RSPs demonstrates that they contain E homodimers and M, indicating that RSPs have trafficked through the secretory pathway and that maturation process has occurred. Using the HeLa-prME cell line, we have developed a semi-quantitative assay to screen a 122 genes cellular membrane trafficking siRNA library and identified genes that may be involved in the secretion of DV. From our screen, 23 genes show a significant reduction in DV1 RSPs secretion whereas for 22 genes RSPs levels were increased in cell supernatants. These data provide a proof of concept that RSPs of DVs and the producing cell line are safe tools that can be used in the study of viral egress. Finally, the optimization strategy was applied to DV2, DV3 and DV4 prME, and RSPs for all four DV serotypes were efficiently produced. These native RSPs are, therefore, a safe mimicry of virions that can be used to study viral and cellular requirements for virus assembly and egress.

## Results

### prME Gene Optimization Significantly Improves Viral Protein Expression in HeLa Cells

**Figure 1:**
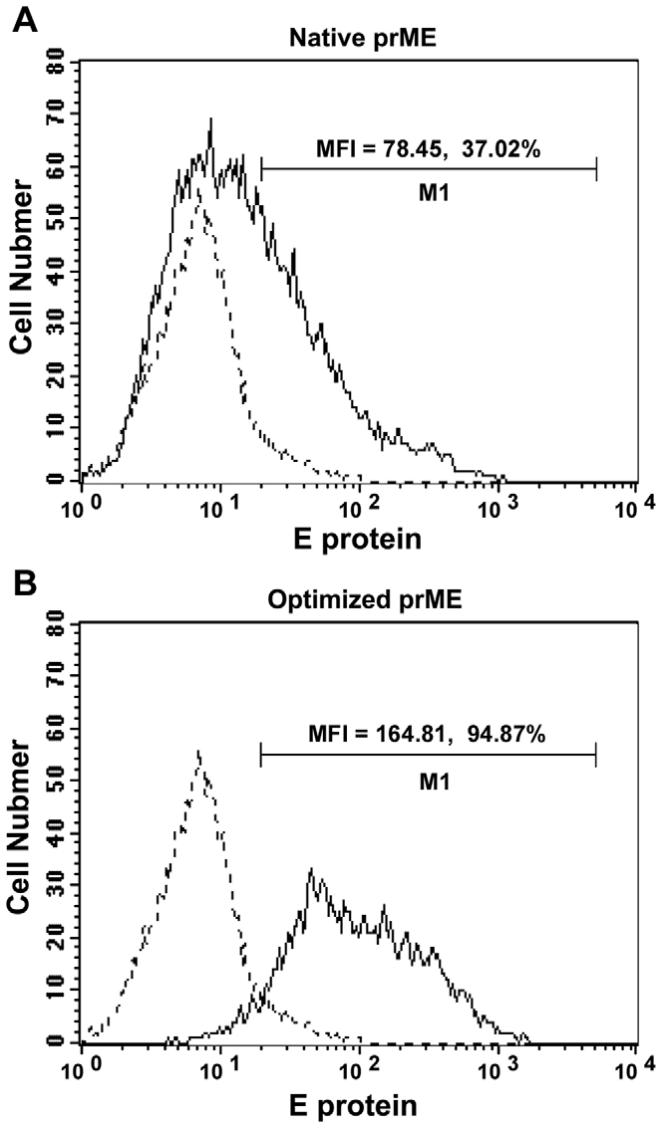
Optimization of codon usage of DV1 prME gene increases its expression level in mammalian cells. HeLa cells were transfected with either native prME gene (A) or prME-opt gene (B). Dotted lines represent the mock control (HeLa cells transfected with pcDNA empty vector). Transfected cells were permeabilized, stained with the 4E11 anti-E monoclonal antibody and FITC-conjugated secondary antibody and analyzed by flow cytometry. The mean fluorescence intensity (MFI) and percentage of positive cells are indicated.

To generate DV RSPs, we first transfected HeLa cells with native DV1 prME gene derived from the DV1 FGA/NA d1d [24] strain, which contained the signal sequence of vesicular stomatitis virus G (VSV-G) envelope glycoprotein inserted in frame upstream of the prME cDNA. 48 hours post-transfection, expression of dengue E protein was monitored by flow cytometry on permeabilized transfected HeLa cells with the 4E11 monoclonal antibody against dengue E protein. E protein was detected in transfected cells at low expression level (Figure 1A). Analysis of the prME gene sequence revealed that 15% of nucleotide triplets were rarely used in mammalian cells so that its codon usage was not adapted for efficient expression in this cell system. Thus we designed and synthesized an optimized DV1 prME (prMEopt) gene in which the rarely used codons were replaced with those preferred by mammalian cells, without changing the amino acid sequence (Figure S1). More than 70% codons were modified for prME optimization. In addition, we also removed the negative cis-acting sites (such as splice sites, poly(A) signals, etc) which might have negatively influenced expression. The optimized gene resulted in high and stable expression levels in transfected mammalian cells (Figure 1B). Both the mean fluorescence intensity (MFI) and percentage of positive cells increased by almost 3 fold. E protein was hardly detected without cell permeabilization, indicating that its localization was intracellular and was not expressed at cell surface (data not shown).

A HeLa cell line that stably expresses DV1 prM and E envelope proteins was then established by transducing HeLa cells with a retroviral vector pCHMWS-prMEopt-IRES-Hygromycin. Hygromycin-selected cells were designated HeLa-prME. Several single colonies of HeLa-prME were used in this study and they showed similar results. The HeLa-prME cell line has already been propagated for more than thirty passages in the presence of hygromycin and has kept a stable prME expression level. Culture of HeLa-prME cells in the absence of hygromycin resulted in a significant reduction of prME expression (data not shown).

### DV1 RSPs Are Found in the Endoplasmic Reticulum Where the E Protein Is Preferentially Localized

**Figure 2:**
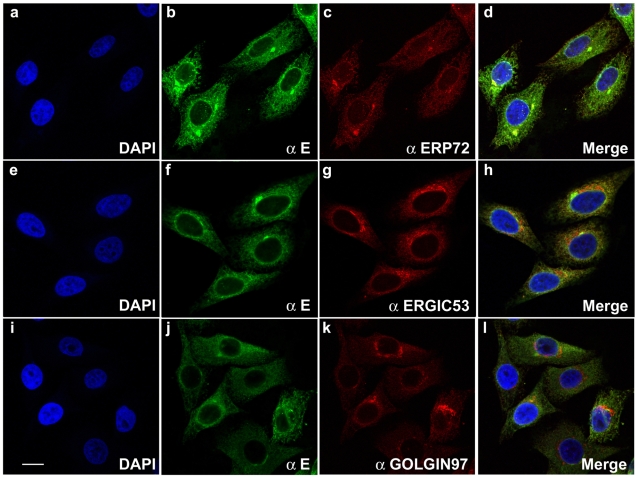
Subcellular localization of the DV1 E protein in HeLa-prME cells. HeLa-prME cells were fixed, permeabilized, and stained for E protein (green) and for cellular marker antigens (red). DAPI staining was used to label cell nuclei (blue). Erp72, ERGIC-53 and Golgin-97 are proteins of the endoplasmic reticulum (ER), the ER-Golgi intermediate compartment (ERGIC) and Golgi apparatus, respectively. The scale bar represents 10 µm.

The intracellular distribution of DV1 E glycoprotein was analyzed in fixed HeLa-prME cells by co-labeling with Erp72, ERGIC-53 and Golgin-97, which are proteins of the endoplasmic reticulum (ER), the ER-Golgi intermediate compartment (ERGIC) and the Golgi apparatus, respectively. As expected, the DV1 E glycoprotein mainly distributed in the ER in HeLa-prME cells, as shown by immunofluorescence, where it colocalized with Erp72 (Figure 2A–D), whereas it showed no colocalization with ERGIC-53 (Figure 2E–H) and Golgin 97 (Figure 2I–L). Interestingly, E and Erp72 were also enriched in a perinuclear compartment, which is not usually labeled by anti-Erp72 antibody and other ER markers (data not shown), suggesting that it has been induced by prM and E viral protein expression. In addition, no staining was observed at the plasma membrane, confirming the previous flow cytometry result. Altogether, our data demonstrate that the DV1 E glycoprotein is efficiently expressed and enriched in the endoplasmic reticulum in the HeLa-prME stable cell line.

**Figure 3:**
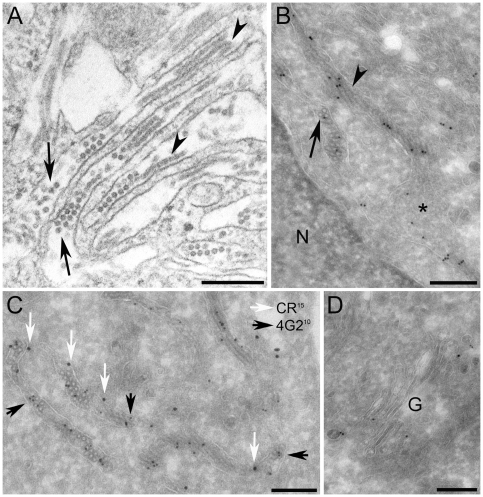
Electron microscopy analysis of the HeLa-prME cell line. HeLa-prME (DV1) cells were fixed and either prepared for epon embedding (A), or for immuno labeling on thawed cryosections (B–D). A), Round particles are found aligned in the lumen of the ER (arrows) together with tubular structures (arrowheads). B) Labeling for the E protein is present in the lumen of cisternae. It is localized to electron lucent, round particles (arrows) and tubular structures (arrowheads). In addition E protein is also found on amorphous, electron dense material present in these cisternae (asterix). C) Double labeling of E protein (10 nm gold, black arrows) and the ER marker calreticulin (15 nm gold, white arrows). Calreticulin is present on the limiting membrane of the cisternae, which contain round particles positive for E protein labeling. D) Label for the E protein is also found within the Golgi stack (G) and on vesicles in the Golgi area. N = nucleus, all scale bars represent 200 nm.

The endoplasmic reticulum is the assembly site for flaviviruses. In order to verify whether this ER staining reflects the presence of E-containing assembled particles, we analyzed the HeLa-prME stable cell line by transmission electron microscopy (TEM) on thin sections of fixed, epon-embedded cells (Figure 3A). We found that HeLa-prME, but not control HeLa cells (data not shown), displayed dilated ER membranes with aligned round particles and elongated parallel tubules. Particles were ∼20 nm in diameter and homogenous in size and shape. The aligned particles that we have observed in HeLa-prME cells are similar to the stacked viral particles which have been described in cisternae of the rough ER in infected insect and mammalian cells [25], [26], [27], [28]. To confirm that these structures contain viral proteins, we labeled thawed cryosections with the 4G2 antibody that recognizes the E protein (Figure 3B–D). We found antibody binding for the E protein present in the lumen of cisternae, where it is localized to electron lucent, round particles and tubular structures. In addition, E protein is also found on amorphous, electron dense material present in these cisternae (Figure 3B). To confirm the nature of these cisternae by other than morphological criteria we performed a double labeling of the 4G2 antibody with the ER resident protein calreticulin. We found antibody binding for calreticulin present on the 4G2 positive cisternae (Figure 3C), unequivocally identifying them as ER. In addition, we found a lower but significant amount of label for the E protein present in the Golgi stack and in vesicles in the Golgi area (Figure 3D).

Thus, our observations indicate that expression of prM and E DV1 envelope proteins induces the formation of RSPs in an ER-derived compartment.

### DV1 RSPs Traffic and Mature through the Secretory Pathway Before Release

**Figure 4:**
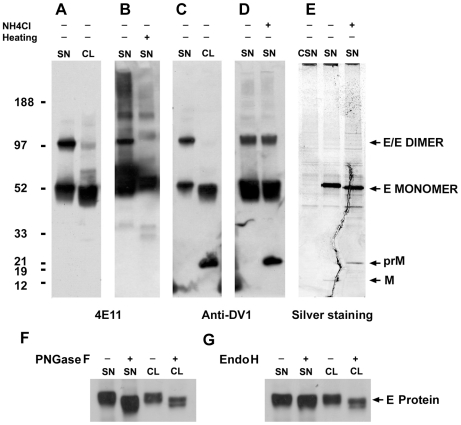
Biochemical analysis of DV1 recombinant subviral particles. The cell lysate (CL) and supernatant (SN) of the HeLa-prME stable cell line were analyzed by Western blot using the 4E11 anti-E monoclonal antibody (A, B, F, G), anti-DV1 mouse IgG (C, D) or by silver-staining (E). A) Detection of E protein in CL and SN of HeLa-prME cell line. B) Homodimer of E could not be detected when samples were heated in presence of dithiothreitol. C) The prM protein could be detected with the anti-DV1 serum in CL but not SN samples. D) In the supernatant of HeLa-prME cells cultured in the presence of NH_4_Cl, the prM protein was also detected. E) The E protein, prM protein and M protein could be observed with silver staining in supernatant from HeLa-prME cell but not in parental HeLa cells (CSN). F, G) E protein in SN bears complex sugar N-glycans. SN and CL samples were treated with PNGase F (panel F) or EndoH (panel G) and subjected to Western blot analysis using 4E11 antibody. E protein in SN but not CL is resistant to EndoH treatment, indicating acquisition of complex sugars in the Golgi apparatus.

We then investigated if the RSPs observed by EM in HeLa-prME cells could undergo maturation and be secreted like DV. Cell lysate (CL) and clarified supernatant (SN) concentrated by ultra-centrifugation were analyzed by Western blotting followed by incubation with either the anti-E monoclonal antibody 4E11 (Figure 4A–B) or an anti-DV1 serum from a human patient (Figure 4C–D). In CL samples, a 50 kDa monomeric form of the E glycoprotein was predominantly observed (Figure 4A,C), whereas high levels of 100 kDa E glycoprotein homodimers were readily detected in SN samples (Figure 4A–D). This suggests that whereas in HeLa-prME cells the majority of viral proteins are localized in the early pre-Golgi secretory pathway in an immature state, secreted RSPs have passed through maturation stages in the Golgi apparatus thus resulting in homodimerization of E proteins. To further confirm the presence of E dimers in SN and exclude the possibility that dimerization resulted from treatment for electrophoresis, the SN were incubated in the presence or absence of a cross linker (3, 3′-dithiobis [sulfosuccinimidylpropionate]; DTSSP) and then subjected to Western blotting using the anti-E 4E11 antibody. Under these conditions, we observed that E dimer/monomer ratio was lower in non-treated RSPs compared to the DTSSP treated (data not shown). These data indicate that, in SN, a significant proportion of E protein is present as a homodimer and detection of E monomers most likely results from partial dissociation under the denaturing conditions of electrophoresis. E homodimers were no longer detected when samples were heated in presence of dithiothreitol (Figure 4B). In addition, the prM protein could be readily detected with the anti-DV1 serum in CL but not SN samples (Figure 4C). By contrast, when the HeLa-prME cells were treated with NH_4_Cl, which inhibits acidification of the trans-Golgi compartment and, hence, the activity of furin protease and prM cleavage, prM was also found in SN (Figure 4D–E). To our surprise E dimers were also detected in these conditions. Interestingly, the E monomers from SN exhibited slower electrophoretic mobility than those of CL samples, which suggests that E glycoproteins have acquired complex N-glycans in the Golgi apparatus. This was confirmed by N-Glycosidase F (PNGase F) and endoglycosidase H (EndoH) digestion (Figure 4 F–G), which cleaves nearly all types of N-glycan chains or only high mannose and some hybrid N-glycan chains from glycoproteins, respectively. Whereas E protein in CL was sensitive to both treatments, only PNGase F digestion increased the electrophoretic mobility of E protein in SN, demonstrating that RSPs had acquired EndoH-resistant, complex N-glycans in the Golgi apparatus before secretion. Moreover, we found that secretion of DV RSPs was altered by incubation of cells at 15°C and 20°C, which block cargo transport from ERGIC to cis-Golgi and exit from the trans-Golgi, respectively, and by brefeldin A treatment, which inhibits exit from ER and induces fusion of Golgi membranes with ER (data not shown). Altogether, homodimerization of E, acquisition of complex sugars and efficient cleavage of prM indicate that prM and E viral proteins have correctly assembled in the ER into RSPs which have trafficked through the secretory pathway before secretion into the cell medium.

### Secretion of DV1 RSPs Assembled from Newly Synthesized Proteins Requires 8 Hours

**Figure 5:**
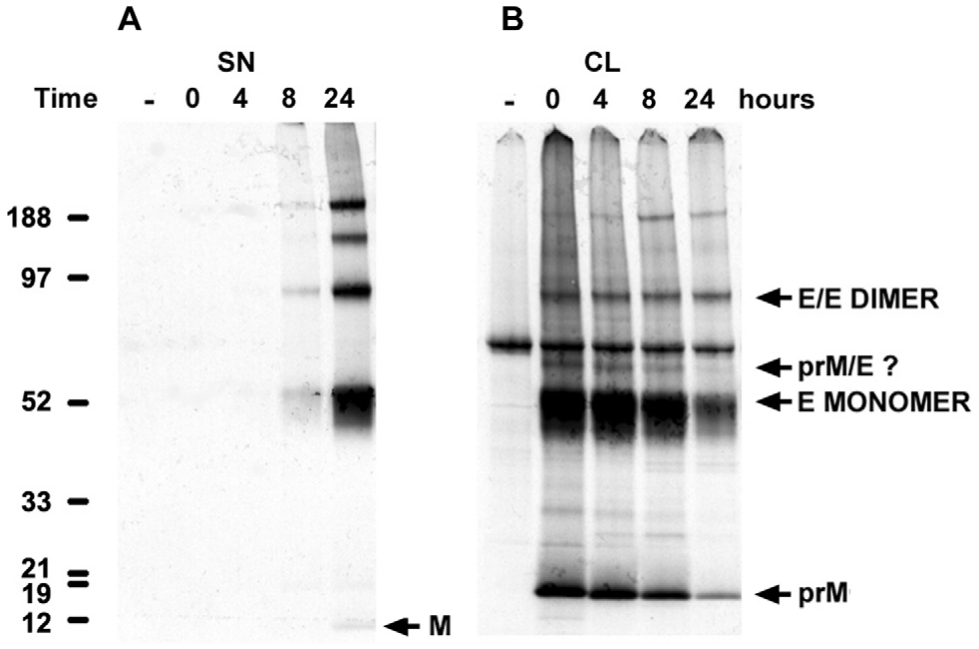
Dynamic study of RSPs secretion. HeLa-prME cells were starved overnight, pulsed with medium containing S^35^-methionine for one hour and then cultured for 0, 4, 8 and 24 hours in complete medium. Cell supernatants (A) and lysates (B) were subjected to immunoprecipitation with anti-E 4E11 antibody. Proteins were separated on SDS-PAGE, and revealed by X-ray autoradiography (3 days exposure).

To study the dynamic of RSP production, we performed a pulse chase experiment on overnight starved HeLa-prME cells that were metabolically labeled with S^35^-methionine for 1 hour and subsequently chased for 24 hours in normal medium. Supernatant and cell lysate were collected at the specified time points after chase; RSPs were immuno-precipitated using the 4E11 anti-E monoclonal antibody and then analyzed by autoradiography. Detection of prM or M in this assay results from co-immunoprecipitation and, therefore, indicates efficient interaction with E protein. Secretion of E and M proteins was detected from the 8 hours time point on, with a significant increase at 24 hours post-chase (Figure 5A). Consistently, high levels of E and M proteins were found in cell lysates at all time points with a substantial diminution at 8 and 24 hours post-chase (Figure 5B). Two major protein products were found in cell lysates, with an apparent molecular weight corresponding to E and prM proteins. Bands of higher molecular weight were also present and could be oligomeric forms of E and prM or M. Interestingly, the pattern of immunoprecipitated proteins from cell supernatant was slightly different. Higher proportions of E dimers were found and a band corresponding to the molecular weight of M but not prM was detected at the 24 hours time point, indicating that most secreted RSPs are mature. Our data demonstrate that newly synthesized proteins need 8 hours to be translocated through the secretory pathway and released into the supernatant as mature RSPs.

### Fractionation of DV1 RSPs on Sucrose Gradient

**Figure 6:**
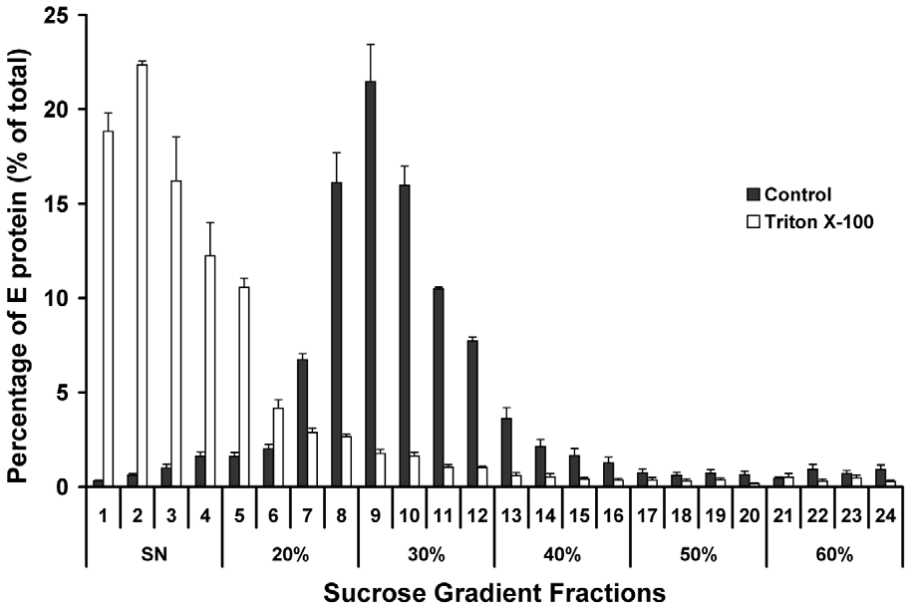
Sucrose gradient analysis of DV1 RSPs. The supernatant from HeLa-prME cells was concentrated and resuspended in PBS or 0.5% Triton-X 100 containing PBS. RSPs were then centrifuged in a 20 to 60% sucrose gradient at 28,000 rpm (Beckman SW-41Ti rotor) for 2.5 hours at 4°C. Fractions of 0.5 ml were collected and E content was measured using CLDB. The percentage of E protein in each fraction is displayed on the Y axis.

To further characterize secreted DV1 RSPs, we performed sucrose gradient fractionation on RSPs concentrated from supernatant of HeLa-prME cells. Supernatants were ultracentrifuged and RSPs-containing pellets either resuspended in PBS or in 0.5% Triton-X100 containing PBS to solubilize E from lipid membranes, as described [29]. As expected, Triton-X100-solubilized E protein and RSP-associated E protein appeared in distinct fractions of a discontinuous 20 to 60% sucrose gradient (Figure 6). The amount of E glycoprotein in each fraction was quantified. E glycoprotein in non-treated sample sedimented in fractions containing 20% to 30% sucrose (Figure 6, black bars) whereas in Triton-X100 treated samples, E protein was solubilized and detected in fractions at the top of the gradient (Figure 6, white bars). These data further confirm that the RSPs formed in HeLa-prME are secreted into culture medium from which they can be easily purified by ultracentrifugation. Moreover, we have obtained preliminary evidence that DV1 RSPs and DV1 viral particles isolated from HeLa-prME and mammalian infected cells, respectively, sediment at similar densities on 10–60% continuous gradient of sucrose (Dr Philippe Despres, Institut Pasteur, personal communication).

### HeLa-prME Cells Producing RSPs Can Be Used to Study the Interaction between DV and Host Cells

**Figure 7:**
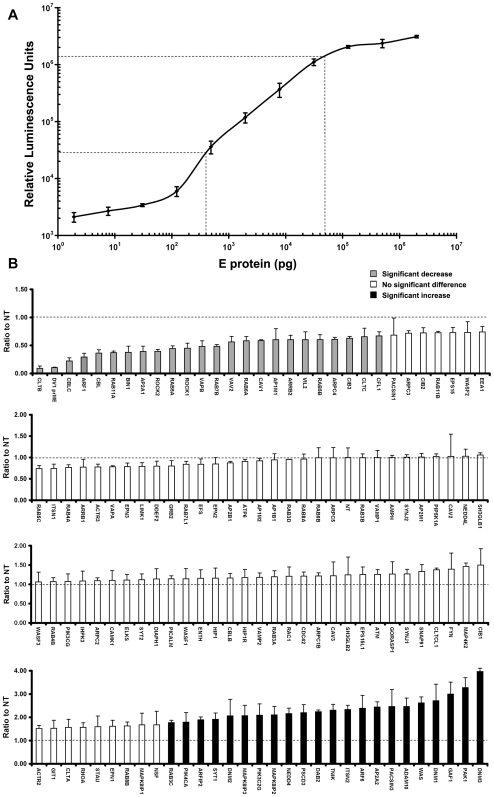
Application of the DV RSP-producing HeLa-prME cell line and CLDB to screen a small library of siRNA which targets 122 genes involved in membrane trafficking. A) The correlation between luminescence density and amount of E protein on PVDF membrane in CLDB assay. B) The screen results of the siRNA library which targets 122 genes involved in membrane trafficking. Non-targeting siRNA (NT) and siRNA targeting DV1 prME were used as controls. Level of E protein in cell supernatant of each siRNA was expressed as its ratio to that of NT controls. Two-sided Student's t test was used to assess the statistical significance of differences between each sample and NT. Differences were considered statistically significant when P<0.05. Genes inducing either a significant decrease or increase in RSPs production are shown in gray and black columns, respectively.

Our results show that the DV1 RSPs produced by HeLa-prME cell line mimic maturation and secretion of DV1, thus providing a useful tool to study the interaction between DV and host cells during viral egress. To identify host factors that could either enhance or reduce production of DV RSPs, we first developed a quantitative assay to relatively quantify levels of secreted particles in supernatant of HeLa-prME cells. The chemiluminescence dot-blot (CLDB) assay is based on the concentration of RSPs from cell supernatant on PVDF membranes, followed by detection of E with a specific horseradish peroxidase (HRP)-conjugated antibody and quantification of substrate-induced luminescence using a luminometer. Our data using purified E protein of known concentrations showed that, when ranging between 400 pg to 40 ng, E protein on PVDF membrane displayed a very good linear correlation with the luminescence density in CLDB assay (Figure 7A). The CLDB was first used to estimate the RSPs yield from HeLa-prME cell line, and we found that the concentration of E protein in supernatant of HeLa-prME cell line was around 500–1000 ng/ml under the culture condition used for siRNA transfection (data not shown). We then screened a siRNA library that consisted of 122 genes which target cellular membrane trafficking using the HeLa-prME cell line. Non-targeting siRNA (NT) and siRNA targeting DV1 prME were added as controls. Library and control siRNAs were transfected in triplicates on 96-well plates. Levels of RSPs secreted by siRNA transfected HeLa-prME were measured by CLDB assay from 40 microliters of supernatant from each well. Levels of E protein in cell supernatant were expressed in relative luminescence units and ratios to that of NT controls were shown in Figure 7B. T test was used to assess the statistical significance of differences between each sample and NT. Differences were considered statistically significant when P<0.05. We observed that targeting of 23 genes resulted in significant reduction of DV1 RSPs amounts in supernatants whereas targeting of 22 other genes induced a significant increase. For instance, our results showed that down-regulation of ADP-ribosylation factor 1 (ARF1), which regulates secretory membrane transport [30], resulted in 3 fold decrease of DV1 RSPs in cell supernatant, suggesting its involvement in the secretion of DV. Some genes whose down-regulation enhanced levels of DV RSPs in the supernatant are involved in endocytosis, such as the three dynamins which show a 2 to 4 fold increase in dengue RSPs secretion. These proteins are involved in the budding process or in the transport of vesicles [31]. Such an enhancement might have been due, at least in part, to a blockade in the re-internalization of secreted RSPs by HeLa-prME cells. Although further experiments are required to confirm the screening data, our study validates the use of DV RSPs-producing HeLa-prME cell line in combination with the CLDB-based quantification strategy as a promising system to facilitate the identification of cellular factors involved in DV secretion.

### Codon Optimization Results in the Efficient Production of DV2, DV3 and DV4 RSPs

**Figure 8:**
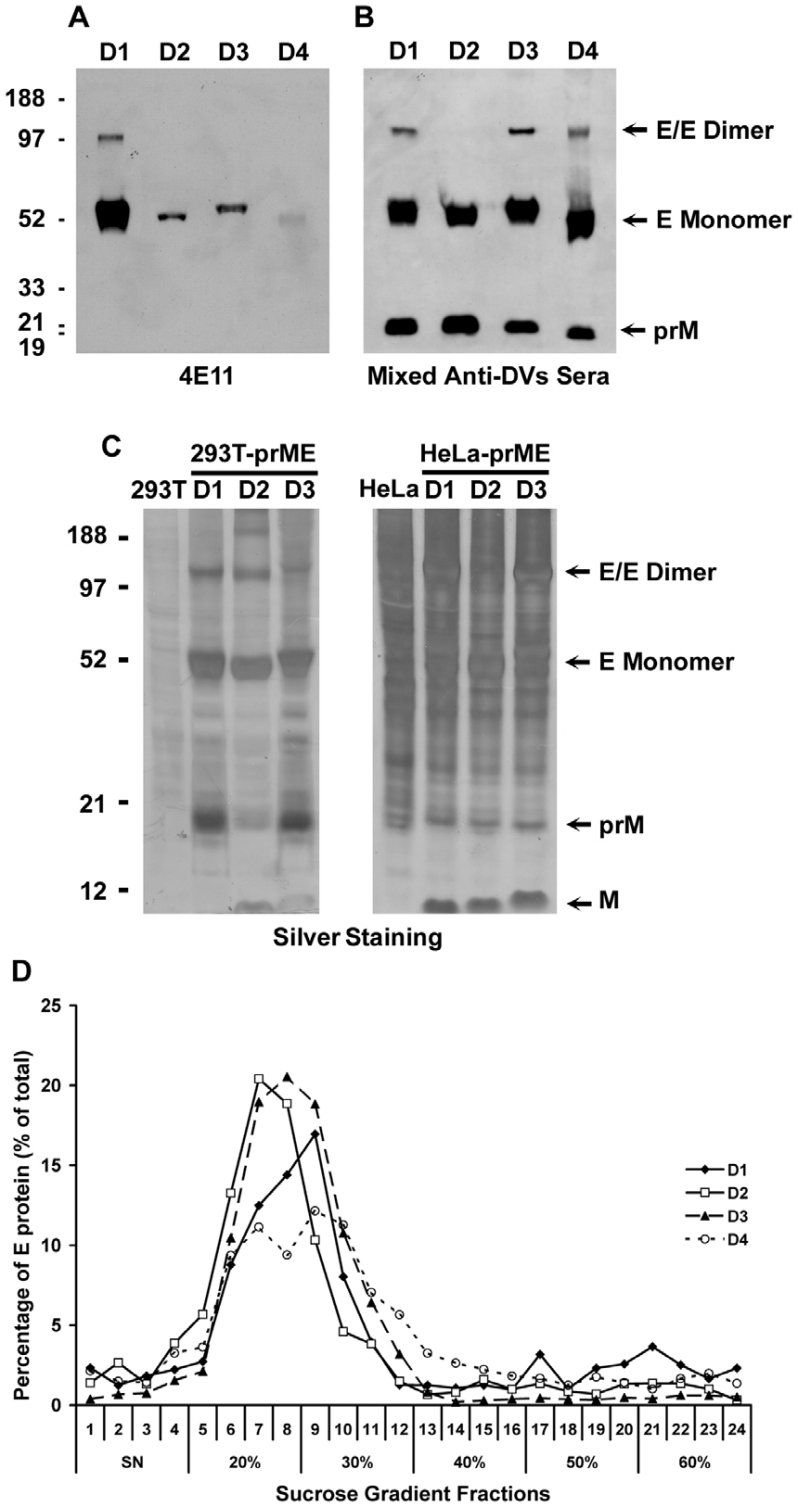
Production of RSPs for all four serotypes of DV. Production of RSPs by 293T cells transiently transfected with optimized prME genes of DV1-DV4. At 48 hours post transfection, the RSPs in supernatant were analyzed by Western blot using anti-E mAb 4E11 (A) or mixture of four sera from patients infected by DV1-DV4, respectively (B). C) Production of RSPs by 293T-prME and HeLa-prME stable cell lines. DV1-DV3 RSPs in supernatants were analyzed by SDS-PAGE and silver staining of polyacrylamide gels. Bands corresponding to the approximate molecular weight of E monomers and dimers, as well as prM and M are indicated by arrows. Supernatants from parental 293T and HeLa cells were used as controls. D) Analysis of RSPs of 4 serotypes by sucrose gradient. RSPs were centrifuged in a 20 to 60% sucrose gradient at 28,000 rpm for 2.5 hours in 4°C. Fractions of 0.5 ml were collected and measured using CLDB. The percentage of E protein in each fraction is displayed on the Y axis.

The codon usage of DV2, DV3 and DV4 prME genes differed substantially from that of mammalian cells and, therefore, was optimized as described for DV1 by replacing more than 70% of the codons (Figure S1). RSPs of the four serotypes were first produced in 293T cells by transient expression of prME genes. Particles were purified and detected by Western blotting using either the anti-E 4E11 monoclonal antibody or a mixture of sera from four patients who were infected by all dengue serotype (Figure 8A–B). With the anti-E 4E11 antibody, monomeric and dimeric forms of the DV1 E protein could be readily detected whereas weaker signals were observed for the other three serotypes, because of limited affinity of the antibody (Figure 8A) [32]. E proteins of all DVs were detected using the mixture of sera (Figure 8B) with a signal of similar intensity, which suggested that RSPs of four serotypes could be generated to comparable levels. Monomeric E proteins of four serotypes displayed slightly different electrophoresis mobility, which could be due to a differential level of N-glycosylation. E glycoproteins of DV1 and DV3 have two N-glycosylation sites at Asn 67 and Asn 153, whereas those of DV2 and DV4 have only one at Asn 67 [33]. Dimeric E protein was observed in DV1, DV3 and DV4 but not in DV2. Besides E, the prM protein was also detected in RSPs produced by transient transfected 293T cells. Recently, we have established HeLa-prME and 293T-prME cell lines of DV1, DV2 and DV3 using the same procedure described for DV1 HeLa-prME. We have compared the maturation of RSPs produced by both cell types using SDS-PAGE and silver staining of the gel (Figure 8C). Supernatants from parental HeLa and 293T cells were used as controls in the experiment. We found that, whereas only a small fraction of prM was cleaved in the RSPs produced by 293T-prME cell lines, cleavage of prM was much more effective in RSPs from the HeLa-prME cell lines. This result indicates that efficacy of maturation is cell type dependent. RSPs of the four serotypes were further analyzed by sucrose gradient. As for DV1, RSPs of DV2, DV3 and DV4 were concentrated in fractions containing 20% to 30% sucrose (Figure 8D). Altogether, our results demonstrated that the strategy of codon optimization was successful in leading to the production of RSPs of all serotypes with comparable efficacy and similar sedimentation properties.

## Discussion

In this study, we have used a codon-optimization strategy to establish and characterize stable cell lines that produce RSPs for the four dengue serotypes. Previous studies had reported the production of DV RSPs in which the glycoproteins were mutated either to avoid host cell fusion or to delete the ER retention signal. For example, DV1 RSPs were not secreted effectively and DV2 RSPs could not form unless the transmembrane domain of the E glycoprotein, which contains an ER retention motif was replaced with that of JEV [22], [23], [34]. Our ability to obtain the efficient production of RSPs of all serotypes without any change in the amino acid sequence is likely the result of codon optimization for expression in mammalian cells, which has been proven to be an effective method to increase the expression level of glycoproteins from various viruses [35], [36]. Thus, gene optimization significantly enhanced expression of prME glycoproteins in transfected cells, and this over-expression increased both RSP production and secretion. However, it is also possible that using other viral strains may result in different efficacy of RSPs production, regardless of codon optimization. To our knowledge, this is the first report of RSPs using the native prM and E envelope proteins for the four serotypes of DVs in mammalian cells.

We have characterized the maturation of DV1 RSPs produced by the HeLa-prME cell line. Sucrose gradient and sedimentation analysis demonstrated that the DV1 RSPs are concentrated in fractions containing 20–30% sucrose and are sensitive to detergent treatment. Although prM/E heterodimers were not found in our experiments, possibly because prM/E interaction is weak, we observed processing of prM into M protein in HeLa-prME cells. This cleavage was sensitive to NH_4_Cl treatment, which inhibits acidification of the trans-Golgi compartment and, hence, the activity of furin protease. Newly synthesized E proteins first form heterodimers with prM proteins in the ER of host cells and then rearrange as homodimers during the process of secretion [37], [38]. The rearrangement from heterodimer to homodimer was first thought to require cleavage of prM by furin in the trans-Golgi to form M and soluble pr proteins [39]. A recent study, however, has shown that such rearrangement is mainly caused by the progressive acidification of the milieu along the secretory pathway, which facilitates prM cleavage by furin [6], [10]. If rearrangement is a pre-requisite for prM cleavage to occur, it could explain, at least in part, why E homodimers were still observed in our study following treatment with the acidotropic reagent NH_4_Cl. The precise mechanism underlying their presence in RSPs under these experimental conditions, however, requires further investigation. Finally, we found by pulse-chase experiments that 8 hours are necessary for production and secretion of RSPs. Altogether, these results show that mature DV1 RSPs are efficiently produced by the stable HeLa-prME cell line.

We have studied the subcellular localization of DV1 E and RSPs in the HeLa-prME cell line. By immuno-staining of fixed cells and fluorescence microscopy, we have shown that most of the E protein is localized in the ER compartment, where dengue glycoproteins are synthesized [1]. The fact that we did not detect any significant signal of E in ERGIC and Golgi apparatus could be explained by a very low amount of protein in these organelles. In these conditions, saturating signals in the ER would mask its detection in other compartments along the secretory pathway. The fluorescent microscopy results were in accordance with our electron microscopy data. Analysis of cell sections by transmission electron microscopy has revealed that E proteins in the ER are associated with both round particles and tubular structures. The tubules could either be intermediate forms of RSPs assembly or result from accumulation of prM and E proteins in the ER. Budding of virions from cellular membranes depends on assembly of viral structural proteins that generate pushing and/or pulling forces simultaneously to induce curvature of membranes, which is necessary for particle formation and membrane fission. It is possible that assembly of overexpressed prM and E proteins in the ER allows formation of long tubules but that the fission event is a limiting factor. The secretion pathway was also investigated by temperature block or pharmacological experiments. Incubation at either 15°C, to stop traffic between the intermediate compartment and the cis-Golgi, or at 20°C, to block exit from the trans-Golgi network, or BFA treatment, to block the exit from ER, significantly reduced secretion of RSPs. These results demonstrate that RSPs traffic through ER, ERGIC and Golgi compartments before being secreted out of the cell.

The efficient production of DV1 RSPs by HeLa-prME cell line and the fact that RSPs could mimic the maturation and secretion processes allowed us setting up an assay to study the interaction between DV and host cells during egress, a step which has received little attention in comparison to DV viral entry and replication [40], [41], [42], [43]. We have validated our assay by using a siRNA library preferentially targeting genes involved in cellular transport. As expected, a number of genes involved in the secretory pathway resulted in reduced release of RSPs in the cell supernatant, whereas other factors were associated with an opposite effect. Clearly, the identification of cellular factors involved in the egress process will be helpful to understand the maturation of DV and its pathogenicity. Moreover, our system will allow comparing the effect of cellular factors using RSPs assembled from the four dengue serotypes to test whether there are strain-specific interactions with host proteins.

## Materials and Methods

### Cell Lines, Anti-DV Antibodies and Constructs

HeLa and 293T cells maintained in our lab [44] were cultured in DMEM containing 10% fetal bovine serum. Purified anti-E 4E11 and 4G2 monoclonal antibodies were provided by Dr. A. Amara and P. Despres (Institut Pasteur, France), respectively. Purified anti-DV1 mouse IgG and sera from four patients infected by the four dengue serotypes (1–4), respectively, were kindly provided by Dr. Philippe Buchy (Institut Pasteur, Cambodia). The native (non-codon optimized) DV1 (strain FGA/NA d1d) prME gene containing construct was provided by Dr A. Amara. The prME sequences of DV1 (strain FGA/NA d1d), DV2 (strain FGA/02), DV3 (strain PAH881/88) and DV4 (strain 63632) were codon-optimized and synthesized by the Geneart Company (Regensburg, Germany) and subcloned into pcDNA or retroviral vector pCHMWS-IRES-Hygromycin (kindly provided by Dr. Rik Gijsbers, from Molecular Medicine at Katholieke Universiteit Leuven) using BamHI and XhoI restriction sites. A nucleotide sequence encoding for the signal peptide of VSV-G (MKCLLYLAFLFIGVNC) was included upstream of each prME gene. Sequences of codon-optimized prME genes are provided as Supporting Information (Figure S1).

To produce the retroviral vector for delivery of the prME-opt gene into HeLa cells, the pCHMWS-prME-opt-IRES-Hygromycin, pcDNA-VSV-G and p8.71 (modified HIV provirus coding for gag and polymerase) plasmids were co-transfected into 293T cells. The cell supernatant containing infectious VSV-G-pseudotyped retroviral particles was harvested 48 hours post-transfection and used to infect HeLa cells. Two days after infection, cells were selected in culture medium containing 500 µg/ml of hygromycin for two weeks. Selected cells (HeLa-prME) were tested by flow cytometry for E expression and were maintained in DMEM +10% FBS +500 µg/ml of hygromycin.

### Flow Cytometry

HeLa cells were transfected with pcDNA-prME, pcDNA-prME-opt or a pcDNA empty vector using calcium phosphate precipitate method. Two days after transfection, cells were detached by incubation in 10 mM EDTA at 37°C for 10 min, fixed in 2% paraformaldehyde, and then permeabilized in 0.1% Triton X-100. After washing, the cells were incubated with a mouse anti-E antibody (4E11, 1∶200) for 1 hour at 4°C. Mouse IgGs (ZYMED, South San Francisco, CA, USA) were used as control. Cells were then washed and incubated for 30 minutes with a goat anti-mouse IgG (H+L) fluorescein isothiocyanate (FITC)-conjugated secondary antibody (1∶200; ZYMED). Cells were analyzed using a Facscalibur flow cytometer (BD Biosciences, San Jose, CA, USA).

### Fluorescence Microscopy

For fluorescence microscopy on fixed cells, HeLa-prME cells were grown on glass coverslips. Cells were fixed, permeabilized, and incubated with anti-DV human sera (1∶200) and anti-Erp72 Rabbit polyclonal Ab (1∶200, Stressgen Bioreagents, Ann Arbor, MI, USA), anti-ERGIC-53 mAb (1∶1000, Alexis Biochemicals, Farmingdale, NY, USA), or anti-Golgin-97 mAb (1∶50, Invitrogen, Carlsbad, CA, USA), followed by the incubation with corresponding secondary antibody conjugated with FITC or TRITC. Nuclei were stained with DAPI and coverslips were mounted on glass slides for analysis. Fixed cells were visualized under AxioObserver Z1 inverted motorized fluorescent microscope using the ApoTome module and piloted through the Axiovision 4.6 software and images were acquired through the MRm AxioCam high resolution CCD camera (Carl Zeiss, Germany).

### Electron Microscopy

HeLa-prME cell line was fixed at 28 hours post-trypsination and processed for EM and immuno-EM.

For conventional TEM, cells were fixed in 2.5% glutaraldehyde in 0.1 M cacodylate buffer (pH 7.2) for 1.5 hr at room temperature and post-fixed with 1% osmium tetroxide in 0.1 M cacodylate buffer (pH 7.2) for 1 hour at room temperature. Then cells were embedded in 2% agarose and cell blocks were post-fixed with 2% uranyl acetate in 30% ethanol, dehydrated in graded series of ethanol and embedded in epoxy resin. Ultrathin sections were cut with a Leica Ultramicrotome UCT (Leica Microsystems; Vienna, Austria) and collected on 400-mesh formvar coated copper grids. Sections were stained 45 minutes with 4% aqueous uranyl acetate and 5 minutes with lead citrate.

For immuno-EM, cells were fixed with 4% formaldehyde in 0.1 M phosphate buffer (pH 7.4), and embedded in 12% gelatin. Blocks were infiltrated with 2.3 M sucrose for cryoprotection, mounted on specimen holders and frozen in liquid nitrogen. Cryosections were cut with a Leica EM UC6/FC6 Microtome (Leica Microsystems, Vienna, Austria). Thawed cryosections were labeled with anti-E 4G2 antibody, rabbit polyclonal antibody against ER resident protein calreticulin (Abcam, Cambridge, MA, USA) and protein-A gold (10 nm and 15 nm) obtained from Utrecht University (Utrecht, The Netherlands) and used as described before [45]. Double labeling was performed sequentially, using for each antibody a different size of protein A gold. Unspecific binding of the second protein A to the first antibody was blocked by incubation with 1% glutaraldehyde as described before [46]. The grids were viewed with Philip CM10 electron microscope at 80 kV and images were taken with KeenView camera (Soft Imaging System, Lakewood, CO, USA) using iTEM 5.0 software (Soft Imaging System GmbH).

### Concentration of RSPs

Supernatants of HeLa-prME or its parent HeLa cells were harvested and cleared by centrifugation at 3,000 rpm for 15 minutes and 10,000 rpm for 30 minutes. Clarified supernatants were then concentrated by ultracentrifugation at 28,000 rpm for 2.5 hours. Pellets were then resuspended in 100 µl of Phosphate buffered saline (PBS). For the production of immature RSPs, the HeLa-prME cells were cultured in medium containing 20 mM of ammonium chloride (NH_4_Cl).

To generate RSPs of dengue 2, 3 and 4, pcDNA constructs containing the optimized prME genes (10 µg each) were transfected into 293T cells separately. The DV1 pcDNA-prME construct was transiently transfected as control. Supernatants of transfected 293T cells were harvested, clarified and concentrated as mentioned above.

### Gel Electrophoresis, Immunoblotting and Silver Staining

After ultracentrifugation, RSPs were resuspended in 100 µl PBS to which 33 µl of 4X NuPAGE LDS (lithium dodecyl sulfate) sample buffer (Invitrogen) was added. RSPs were then analyzed by sodium dodecyl sulfate-polyacrylamide gel electrophoresis (SDS-PAGE) using 4∼12% NuPAGE Novex Bis-Tris Gels (Invitrogen). For analysis of cell lysates by SDS-PAGE, HeLa-prME cells were detached by incubation in 10 mM EDTA at 37°C for 10 min and resuspended in 500 µl of PBS. Cell suspensions were then mixed with 167 µl 4X of sample buffer and then sonicated before electrophoresis of samples as described above. For immunodetection, proteins were blotted from gels onto polyvinylidene difluoride (PVDF) membranes. The membrane was blocked overnight in 5% milk in PBST solution and then incubated with anti-E antibody (4E11, 1∶1000) for 1 hour. After washes, the membrane was incubated for 1 hour at room temperature with a horseradish peroxidase-labeled goat anti mouse IgG polyclonal antibody. The membrane was finally visualized using ECL Western blot detection reagents (Invitrogen) and Amersham Hyperfilm ECL (GE Healthcare, Waukesha, WI, USA). For silver staining, the gel was fixed for 30 minutes and incubated with sodium thiosulfate for 30 minutes at room temperature. After three washes, the gel was incubated with Silver Nitrate for 40 minutes and developed for 15 minutes in sodium carbonate solution (25 g/L). EDTA solution (40 mM) was used to stop the development.

For the endoglycosidase treatment, RSPs or HeLa-prME cell lysates were treated with 500 U of EndoH or PNGase F at 37°C for 3 hours according to the manufacturer's instructions (New England Biolabs, Beverly, MA, USA), and subsequently analyzed by Western blot.

### Relative Quantification of RSPs by Chemiluminescence Dot-Blot

A chemiluminescence dot-blot (CLDB) method was developed to quantify RSPs. Briefly, 40 µl of supernatant of either HeLa-prME cell line or purified E protein solution at known concentration were blotted onto PVDF membrane through a Dot Blot 96 System (Biometra, Goettingen, Germany). The membrane was blocked overnight in 5% milk in PBST solution, incubated with anti-E antibody (4E11, 1∶10,000) for 1 hour and then for 1 additional hour with a peroxidase-labeled goat anti mouse IgG polyclonal antibody (1∶10,000; ZYMED). ECL Western blot detection reagents (Invitrogen), diluted five times, were mixed and added to the membrane and the luminescence intensity was measured using the Microbeta luminometer (PerkinElmer, Waltham, MA, USA).

### Sucrose Gradient

For sucrose gradient analysis, concentrated RSPs were ultra-centrifuged in a 20 to 60% discontinuous sucrose gradient at 28,000 rpm (Beckman SW-41Ti rotor) for 2.5 hours in 4°C. All sucrose solutions were prepared with HEPES buffer (20 mM). Fractions of 0.5 ml were collected and levels of E were measured using the CLDB assay. Alternatively, RSPs were treated with 0.5% Triton X-100 for 1 hour before sucrose gradient fractionation, for RSPs denaturation.

### Screen of the siRNA Library and Statistical Analysis

The human membrane trafficking siRNA library targeting 122 genes and the corresponding transfection reagents were purchased from Dharmacon (#G-005500; Dharmacon Research Inc, Lafayette, CO, USA). For the screen, the HeLa-prME cells were seeded in eight 96-well plates with 10,000 cells per well. 24 hours later, 10 pmol of each siRNA was added to each well together with the transfection reagent. siRNA targeting DV1 prME (target sequence: AGATCCAGCTGACCGATT) and non-targeting siRNA (NT) (Dharmacon Research Inc) were used as controls. Each plate contained in triplicates 15–16 siRNAs from the library, as well as positive (DV1 prME) and negative (NT) siRNA controls. Culture medium was changed two days post-transfection, the supernatant from each well was harvested after an additional 48 hour incubation and then cleared by centrifugation at 4000 rpm for 15 min. 40 µl of supernatant from each well were used to measure by CLDB the levels of RSPs secreted by siRNA transfected HeLa-prME. Two-sided Student's t test was used to assess the statistical significance of differences between each sample and the non-targeting control. Differences were considered statistically significant when P<0.05. Levels of E protein in cell supernatant were expressed in relative luminescence units and ratios of experimental conditions to controls, set as unity, were calculated.

## Supporting Information

Figure S1 The four optimized DV prME sequences. Each optimized prME gene has a BamH I restriction enzyme site, a kozak sequence GCCACC, a signal sequences from VSV-G, and a Xho I restriction enzyme site.
(0.03 MB DOC)
