# Supplementary material for: Efficient Assembly and Secretion of Recombinant Subviral Particles of the Four Dengue Serotypes Using Native prM and E Proteins
Source: PLoS One. 2009 Dec 15;4(12):e8325. doi: 10.1371/journal.pone.0008325 (PMC2790604; doi:10.1371/journal.pone.0008325)
Supplement: Figure S1 — The four optimized DV prME sequences. Each optimized prME gene has a BamH I restriction enzyme site, a kozak sequence GCCACC, a signal sequences from VSV-G, and a Xho I restriction enzyme site. (0.03 MB DOC) [file pone.0008325.s001.doc]

**DV1 opt prME:**

GGATCCGCCACCATGAAGTGCCTGCTGTACCTGGCCTTCCTGTTCATCGGCGTGAACTGCTTCCACCTGACCACCAGAGGCGGCGAGCCCCACATGATCGTGTCCAAGCAGGAGAGAGGCAAGAGCCTGCTGTTCAAGACCAGCGCCGGAGTGAACATGTGTACCCTGATCGCCATGGATCTGGGCGAGCTGTGTGAGGACACCATGACCTACAAGTGCCCCAGAATCACCGAGGCCGAGCCCGACGACGTGGACTGCTGGTGTAACGCCACCGATACCTGGGTGACCTACGGCACCTGTAGCCAGACCGGCGAGCACAGGAGAGACAAGAGAAGCGTGGCCCTGGCCCCCCATGTGGGCCTGGGCCTGGAGACCAGAACCGAGACCTGGATGAGCAGCGAGGGCGCCTGGAAGCAGATCCAGAAGGTGGAGACCTGGGCCCTGAGACACCCCGGCTTCACCGTGATCGCCCTGTTCCTGGCCCACGCCATCGGCACCAGCATCACCCAGAAGGGCATCATCTTCATCCTGCTGATGCTGGTGACCCCTAGCATGGCCATGAGATGTGTGGGCATCGGCAACAGGGACTTCGTGGAGGGCCTGAGCGGCGCCACCTGGGTGGACGTGGTGCTGGAGCACGGCAGCTGTGTGACCACCATGGCCAAGAACAAGCCCACCCTGGACATCGAGCTGCTGAAAACCGAGGTGACCAACCCTGCCGTGCTGAGGAAGCTGTGTATCGAGGCCAAGATCAGCAACACCACCACCGACAGCAGATGCCCCACCCAGGGCGAGGCCACCCTGGTGGAGGAGCAGGACGCCAACTTCGTGTGTCGGAGGACCGTGGTGGACAGAGGCTGGGGCAACGGCTGTGGCCTGTTCGGCAAGGGCAGCCTGCTGACCTGTGCCAAGTTCAAGTGTGTGACCAAGCTGGAGGGCAAGATCGTGCAGTACGAGAACCTGAAGTACAGCGTGATCGTGACCGTGCACACCGGCGACCAGCACCAAGTGGGCAACGAGACCACCGAGCACGGCACCATCGCCACCATCACCCCCCAGGCCCCTACCAGCGAGATCCAGCTGACCGATTACGGCACCCTGACCCTGGATTGTAGCCCTAGAACCGGCCTGGACTTCAACGAGATGGTGCTGCTGACCATGAAGGAGAAGAGCTGGCTGGTGCACAAGCAGTGGTTCCTGGACCTGCCCCTGCCCTGGACCAGCGGCGCCAGCACCTCCCAGGAGACCTGGAACAGACAGGACCTGCTGGTGACATTCAAGACCGCCCACGCCAAGAAGCAGGAGGTGGTGGTGCTGGGCAGCCAAGAGGGCGCCATGCACACCGCCCTGACAGGCGCCACCGAGATCCAGACCAGCGGCACCACCACAATCTTCGCCGGCCACCTGAAGTGTCGGCTGAAGATGGACAAGCTGACCCTGAAGGGCATGAGCTACGTGATGTGTACCGGCAGCTTCAAGCTGGAGAAGGAGGTGGCCGAGACCCAGCACGGCACAGTGCTGGTGCAGGTGAAGTACGAGGGCACCGACGCCCCCTGTAAGATCCCTTTCAGCACCCAGGATGAGAAGGGCGTGACACAGAACGGCAGACTGATCACCGCCAACCCCATCGTGACCGACAAGGAGAAGCCCGTGAACATCGAGACCGAGCCCCCCTTCGGCGAGAGCTACATCATTGTGGGAGCCGGCGAGAAGGCCCTGAAGCTGTCCTGGTTCAAGAAGGGCAGCAGCATCGGCAAGATGTTCGAGGCCACCGCCAGAGGCGCCAGAAGAATGGCCATCCTGGGCGATACCGCCTGGGACTTCGGCTCCATCGGCGGCGTGTTCACCTCTGTGGGCAAGCTGGTGCATCAGGTGTTCGGCACCGCCTACGGCGTGCTGTTCAGCGGAGTGAGCTGGACCATGAAGATCGGCATCGGCATCCTGCTGACATGGCTGGGCCTGAATTCTAGAAGCGCCAGCCTGAGCATGACCTGTATCGCTGTGGGCATGGTGACCCTGTACCTGGGCGTGATGGTGCAGGCCTGATGACTCGAG

**DV2 opt prME:**

GGATCCGCCACCATGAAATGTCTGCTGTACCTGGCCTTCCTGTTCATCGGCGTGAATTGTTTCCACCTGACCACCAGGAACGGCGAGCCCCACATGATCGTGAGCAGACAGGAGAAGGGCAAGAGCCTGCTGTTCAAGACCGAGGACGGCGTGAACATGTGTACCCTGATGGCCATGGACCTGGGCGAGCTGTGCGAGGACACCATCACCTACAAGTGTCCCTTCCTGAGGCAGAACGAGCCCGAGGACATCGACTGTTGGTGTAACAGCACAAGCACCTGGGTGACCTACGGCACCTGCACCACCACCGGCGAGCACAGAAGGGAGAAGAGGAGCGTGGCCCTGGTGCCCCACGTGGGCATGGGATTGGAAACCAGAACCGAGACCTGGATGAGCAGCGAGGGCGCTTGGAAGCATGCCCAGAGAATCGAGACCTGGATTCTGAGACACCCCGGCTTCACCATCATGGCCGCCATCCTGGCCTACACCATCGGCACCACCCACTTCCAGAGAGCCCTGATCTTCATCCTGCTGACCGCCGTGGCCCCCAGCATGACCATGAGATGCATCGGCATCAGCAACAGAGACTTCGTGGAGGGCGTGAGCGGCGGCAGCTGGGTGGACATCGTGCTGGAGCACGGCAGCTGTGTGACCACCATGGCCAAGAACAAGCCCACACTGGACTTCGAGCTGATCAAGACCGAGGCCAAGCAGCCCGCCACCCTGAGAAAGTACTGTATCGAGGCCAAGCTGACCAACACCACCACCGACAGCAGATGCCCCACCCAGGGCGAGCCCAGCCTCAATGAGGAGCAGGACAAGAGATTCGTGTGTAAGCACAGCATGGTGGACAGAGGCTGGGGCAACGGCTGTGGCCTGTTCGGCAAGGGCGGCATCGTGACCTGTGCCATGTTCACATGCAAGAAGAACATGAAGGGCAAGGTGGTGCAGCCTGAGAACCTGGAGTACACCATCGTGATCACCCCTCACTCTGGCGAGGAGCATGCCGTGGGCAACGACACCGGCAAGCACGGCAAGGAGATCAAGATCACCCCCCAGAGCAGCATCACAGAGGCCGAGCTGACCGGCTACGGCACAGTGACCATGGAGTGTAGCCCTAGAACCGGCCTGGATTTCAACGAGATGGTGCTGCTGCAAATGGAGAACAAGGCCTGGCTGGTGCACAGACAATGGTTCCTGGATCTGCCTCTGCCCTGGCTGCCTGGCGCCGACACCCAGGGAAGCAACTGGATTCAGAAGGAGACCCTGGTGACCTTCAAGAACCCCCACGCCAAGAAGCAGGACGTGGTGGTGCTGGGCAGCCAGGAGGGCGCCATGCACACCGCCCTGACAGGCGCCACCGAGATCCAGATGAGCAGCGGCAACCTGCTGTTCACCGGCCATTTGAAATGTAGACTGAGAATGGATAAGCTGCAGCTGAAGGGCATGTCTTACAGCATGTGTACAGGCAAGTTCAAGGTGGTGAAGGAGATCGCCGAGACCCAGCACGGCACCATCGTGATCAGAGTGCAGTACGAGGGCGATGGCAGCCCCTGTAAGATCCCCTTCGAGATCATGGATTTGGAGAAGAGACACGTGCTGGGCAGACTGATCACCGTGAACCCCATCGTGACCGAGAAGGATAGCCCCGTGAACATCGAGGCCGAGCCCCCTTTCGGCGACAGCTACATCATCATCGGCGTGGAGCCCGGCCAGCTGAAGCTGAACTGGTTCAAGAAGGGCAGCAGCATCGGCCAGATGATCGAGACCACCATGAGAGGAGCCAAGCGGATGGCCATCCTGGGCGACACCGCCTGGGACTTCGGCTCTCTGGGCGGCGTGTTCACCTCCATCGGCAAGGCCCTGCACCAGGTGTTCGGCGCCATCTACGGCGCCGCCTTCTCCGGCGTGTCCTGGACCATGAAGATCCTGATCGGCGTGATCATCACCTGGATCGGCATGAATTCCAGAAGCACCAGCCTGAGCGTGTCCCTGGTGCTGGTCGGAGTGGTGACCCTGTACCTGGGCGTGATGGTGCAGGCCTGATGACTCGAG

**DV3 opt prME:**

GGATCCGCCACCATGAAGTGTCTGCTGTACCTGGCCTTCCTGTTCATCGGCGTGAACTGCTTCCATCTGACCAGCAGAGACGGCGAGCCCAGAATGATCGTGGGCAAGAACGAGAGAGGCAAGAGCCTGCTGTTCAAGACCGCCAGCGGCATCAACATGTGTACCCTGATCGCCATGGACCTGGGCGAGATGTGTGACGACACCGTGACCTACAAGTGTCCCCACATCACCGAGGTGGAGCCCGAGGACATCGACTGTTGGTGTAACCTGACAAGCACCTGGGTGACCTACGGCACATGCAACCAGGCCGGCGAGCACAGAAGGGACAAGAGAAGCGTGGCCCTGGCCCCCCACGTGGGCATGGGCCTGGATACCAGAACCCAGACCTGGATGAGCGCCGAGGGCGCTTGGAGACAGGTGGAGAAGGTGGAGACCTGGGCCCTGAGACACCCCGGCTTCACCATCCTGGCCCTGTTCCTGGCCCATTACATCGGCACCAGCCTGACCCAGAAGGTGGTGATCTTCATCCTGCTGATGCTGGTGACCCCCAGCATGACCATGCGGTGTGTGGGCGTGGGCAACAGAGATTTCGTGGAGGGCCTGAGCGGCGCCACCTGGGTGGACGTGGTGCTGGAGCACGGCGGCTGTGTGACCACCATGGCCAAGAATAAGCCCACCCTGGATATCGAGCTGCAGAAGACCGAGGCCACCCAGCTGGCCACCCTGAGAAAGCTGTGCATCGAGGGCAAGATCACCAACATCACCACCGACAGCCGGTGTCCTACCCAAGGCGAGGCCATCCTGCCCGAGGAGCAGGACCAGAACTACGTGTGCAAGCACACATATGTGGATAGAGGCTGGGGCAACGGCTGTGGCCTGTTCGGCAAGGGCAGCCTGGTGACCTGTGCCAAGTTCCAATGTCTGGAGTCTATCGAGGGCAAGGTGGTGCAGCACGAGAACCTGAAGTACACCGTGATTATCACCGTGCACACCGGCGACCAGCACCAGGTGGGCAACGAGACCCAGGGCGTGACCGCCGAGATCACCTCCCAGGCCAGCACAGCCGAGGCCATCCTGCCCGAGTACGGCACCCTGGGCCTGGAGTGTTCCCCCAGGACCGGCCTGGACTTCAATGAGATGATCCTGCTGACCATGAAGAACAAGGCTTGGATGGTGCACAGACAGTGGTTCTTCGACCTGCCCCTGCCCTGGACCAGCGGCGCCACCACCAAGACCCCCACATGGAACAGAAAGGAGCTGCTGGTGACCTTCAAGAACGCCCACGCCAAGAAGCAGGAGGTGGTGGTGCTGGGCAGCCAAGAGGGCGCCATGCACACCGCCCTGACCGGCGCCACCGAGATCCAGACCAGCGGCGGCACATCTATCTTCGCCGGCCACTTGAAATGTAGACTGAAGATGGACAAGCTGAAGCTGAAGGGCATGAGCTACGCCATGTGCCTGAACACCTTCGTGTTGAAAAAGGAGGTGAGCGAGACCCAGCACGGCACCATCCTGATCAAGGTGGAGTACAAGGGCGAGGACGCCCCCTGTAAGATCCCTTTCAGCACCGAGGATGGCCAGGGCAAGGCCCACAACGGCAGACTGATCACCGCCAACCCCGTGGTGACCAAGAAGGAGGAGCCCGTGAACATCGAGGCCGAGCCCCCCTTCGGCGAGAGCAACATCGTGATCGGCATCGGCGACAAGGCCCTGAAGATCAACTGGTACAGAAAGGGCAGCAGCATCGGCAAGATGTTCGAGGCCACCGCCAGGGGCGCCAGAAGGATGGCTATCCTGGGCGACACCGCCTGGGACTTCGGCAGCGTGGGCGGCGTGCTGAACAGCCTGGGCAAGATGGTGCACCAGATCTTCGGCAGCGCCTACACAGCCCTGTTCTCCGGAGTGAGCTGGATCATGAAGATCGGAATCGGCGTGCTGCTGACCTGGATCGGACTGAATTCCAAAAACACCAGCATGAGCTTCAGCTGTATCGCCATCGGCATCATCACCCTGTACCTGGGAGTGGTGGTGCAGGCCTGATGACTCGAG

**DV4 opt prME:**

GGATCCGCCACCATGAAGTGTCTGCTGTACCTGGCCTTCCTGTTCATCGGCGTGAACTGTTTCAGCCTGAGCACCAGAGACGGCGAGCCCCTGATGATCGTGGCCAAGCACGAGAGAGGCAGACCCCTGCTGTTCAAGACCACCGAGGGCATCAACAAGTGTACCCTGATTGCCATGGATCTGGGCGAGATGTGTGAGGACACCGTGACCTACAAGTGTCCCCTGCTGGTGAACACAGAGCCCGAGGACATCGACTGCTGGTGTAACCTGACCAGCACATGGGTGATGTACGGCACCTGTACCCAGAGCGGCGAGAGGAGACGGGAGAAGAGATCCGTGGCCCTGACCCCTCACAGCGGCATGGGCTTGGAAACAAGGGCTGAGACCTGGATGAGCAGCGAGGGCGCCTGGAAGCACGCCCAGAGAGTGGAGAGCTGGATTCTGAGAAACCCTGGCTTCGCCCTGCTGGCCGGCTTCATGGCCTACATGATCGGCCAGACCGGCATCCAGAGGACCGTGTTCTTCGTGCTGATGATGCTGGTCGCCCCCAGCTACGGCATGAGATGTGTGGGCGTGGGCAACCGGGACTTCGTGGAGGGCGTGAGCGGCGGCGCCTGGGTGGACCTGGTGCTGGAGCACGGCGGCTGTGTGACCACCATGGCCCAGGGCAAGCCTACACTGGACTTCGAGCTGACCAAGACCACCGCCAAGGAGGTGGCCCTGCTGAGAACCTACTGTATCGAGGCCAGCATCAGCAACATCACCACCGCCACCAGGTGTCCCACCCAAGGCGAGCCTTATTTGAAAGAAGAGCAGGACCAGCAGTACATTTGTAGAAGAGACGTGGTGGACAGAGGCTGGGGCAACGGCTGTGGCCTGTTCGGCAAGGGCGGCGTGGTGACCTGCGCCAAGTTCAGCTGTAGCGGCAAGATCACCGGCAACCTGGTGCAGATCGAGAACCTGGAGTACACCGTGGTGGTGACCGTGCACAACGGCGATACCCACGCCGTGGGCAACGATACCAGCAACCACGGCGTGACCGCCATGATCACCCCCAGAAGCCCCAGCGTGGAGGTGAAGCTGCCCGACTACGGCGAGCTGACCCTGGACTGCGAGCCCAGAAGCGGCATCGACTTCAACGAGATGATTTTGATGAAAATGAAAAAGAAGACCTGGCTGGTGCACAAGCAATGGTTCCTGGACCTGCCCCTGCCCTGGACCGCCGGAGCCGACACCAGCGAGGTGCACTGGAATTACAAGGAGAGAATGGTGACCTTCAAGGTGCCCCACGCCAAGAGACAGGACGTGACCGTGCTGGGCAGCCAGGAGGGCGCCATGCACAGCGCCCTGGCCGGCGCCACCGAGGTGGATAGCGGCGACGGCAACCATATGTTCGCCGGCCACTTGAAATGCAAAGTGAGGATGGAGAAGCTGAGAATCAAGGGCATGTCCTACACAATGTGTAGCGGCAAGTTCAGCATCGATAAGGAGATGGCCGAGACCCAGCATGGCACCACCGTGGTGAAGGTGAAGTACGAGGGCGCCGGCGCTCCCTGCAAGGTGCCCATTGAGATCAGAGACGTGAACAAGGAGAAGGTGGTGGGCAGAATCATCAGCTCTACCCCCCTGGCCGAGAACACCAATAGCGTGACCAACATCGAGCTGGAGCCTCCCTTCGGAGACAGCTACATCGTGATCGGCGTGGGCAACAGCGCCCTGACCCTGCACTGGTTCAGAAAGGGCAGCTCTATCGGCAAGATGTTCGAGTCCACATATAGAGGCGCCAAGAGAATGGCCATCCTGGGCGAGACCGCCTGGGATTTCGGCAGCGTGGGCGGCCTGTTCACCAGCCTGGGCAAGGCCGTGCACCAGGTGTTCGGCTCCGTGTACACCACCATGTTCGGCGGCGTGAGCTGGATGATCCGGATTCTGATCGGCTTCCTGGTGCTGTGGATCGGCACGAATTCCAGAAACACCAGCATGGCCATGACCTGTATCGCCGTGGGCGGAATCACCCTGTTCCTGGGATTCACCGTGCAGGCCTGATGACTCGAG
